# Protecting Our Fledgling Families: A Case for Relationship-Focused Family Life Education Programs

**DOI:** 10.4103/0970-0218.69246

**Published:** 2010-07

**Authors:** Jane A Henry

**Affiliations:** Department of Psychiatric Social Work, National Institute of Mental Health and Neuro-Sciences, Bangalore, Karnataka, India

The family is a means through which society has been extending itself and ensuring its own existence. In India, marriage is the institution through which society provides legitimacy and ensures the smooth functioning of a family. Societal sanctions and norms are transmitted through family members from generation to generation with various additions and deductions according to the belief systems of the era in which the society exists.

## Transitions in the Indian Family

More recent crises in Indian families encompass many of the same kinds of problems that have plagued countries in the West at least since the 1960s. These include, e.g., marital strain and dissolution, parent–child conflicts and various forms of family violence. Given these conditions and difficulties the future and well-being of the Indian family is uncertain.(1,2)

Subtle changes in family patterns especially with regard to the use of authority within the family as well as an increased focus on individual autonomy(3,4) are also likely to influence members’ expectations of marriage and their choice of marriage partner. Educated middle class families are now more hesitant to make decisions for their offspring with regard to marriage, education, and employment.(4) Changes have also been noticed with regard to a greater focus on the husband”wife relationship rather than the parent–child relationship. With an increased onus of responsibility falling on the individual rather than on the entire family, young Indian adults today face what Dr. Gore calls “choice anxiety” – increased autonomy and increased choice that have led to increased anxiety.(4)

An increase in single-parent families has contributed to a greater percentage of children and adolescents experiencing serious difficulties. In total, increased role strain, marital difficulties, parent–child conflicts, feelings of guilt, and status confusion are commonly observed among working women, even though there are some economic and self-esteem-related advantages. Since more women in India are joining the labor force without proper support and assistance often in the face of extended family and community opposition, an increase in family difficulties is to be expected.(5–8)

## Family Life Education

Family life education seeks to bridge the gap between increasingly fast-paced societies and the traditional values, skills, and knowledge on family living that have kept these societies from disintegrating. The task of passing on education in family living to the next generation has been part of the human experience since life began. But how and by whom it is done have varied among family groups, tribes, and cultures. It has also drastically changed over time and is still evolving. However, at the beginning of the 21^st^ century, one thing is clear – the family in all its forms and circumstances needs support and education. Family life education is being increasingly seen as the answer to this need.

In the 1960s and early 70s with the dawn of the “Woodstock Generation,” United States was faced with the erosion of the institution of marriage, rising divorce rates, increased number of single-parent homes, teen pregnancy, increased incidence of sexually transmitted diseases, and high-risk sexual behavior among teenagers. As a counteractive measure, the impartation of education was taken up as a preventative tool. Family life education in the United States began to embrace disciplines and professionals from the fields of health, education, sociology, psychology, anthropology, law, medicine, biology, child development, and family studies.

Canada has also seen great activity in the area of family life education in the last 40 years. In 1964, the first Canadian Conference on the Family brought together leaders in the family area.(9) Out of this conference was born the Vanier Institute of the Family, in order to “encourage research and study in family life as well as serve as a clearing house for information.”(9) In 1993, the Canadian Certified Family Educator was established.

Currently in the West, family life education as a preventative tool is used extensively with family members in varied stages of the family life cycle to equip them with the necessary skills that they would require to carry out the varied developmental tasks at each life- cycle stage.

## Marital Life Education

A very integral part of the family life education has been marriage education. The belief that satisfying, stable marriages are advantageous to not only partners but also to their offspring is reflected in a growing consensus among researchers, theoreticians, and policy makers that children who grow up in a healthy, happy, stable marriage tend to do better in life.(10,11) The strong link between marital functioning and a wide range of adult and child outcomes have led to a growing recognition among researchers and policy makers that healthy marriages have important public health consequences.(12–14)

Marital life education covers two basic types of approaches to education for relationships and marriage: marriage preparation and marriage enrichment. Marital preparation is designed to help couples before they marry to face marriage realistically and to identify and begin to address specific issues in their own personal lives. Marriage enrichment is a generic term that refers to preventive programs designed to help couples in functioning marriages increase their relationship satisfaction and avoid marital breakdown.

Globally we also see the encouragement and funding of couple relationship education strongly supported in various countries such as Australia, Germany, Singapore, the United Kingdom, and the United States.(12,15,16)

## Marital and Family Life Education in India

In contemporary India, relationship-focused family life education usually operates through informal channels of education (such as from grandparents, uncles, aunts, big brothers, and sisters, i.e., mostly individuals in the joint family system). The Indian family faces issues not so much about teenage pregnancies and divorce as much as issues of a burgeoning population, lack of proper hygiene, malnutrition, inadequate mother and child care, the lack of knowledge, and misconceptions about sexual functioning.

The Family Planning Association of India (FPAI) was the first government body to bring a family life education program for the young in India as early as the late 60s.(17) Currently the FPAI has specialized services and training in family life, marriage, and sex counseling through 36 Sex Education, Counseling, Research, Training/Therapy (SECRT) Centers.(18) According to the guidelines, family life education should start in class 6 and continue till class 12.

The focus of most family life education programs in India has been on sexually transmitted diseases,(17) nutrition and hygiene,(17) reproductive health, and contraception.(19) India’s policies on family and child welfare have been focused more in terms of health care, nutrition, reproductive health, sexuality, and venereal disease. However, one cannot forget that young adults of marriageable age from “first-generation urban educated families”(4) are facing relationship-based problems due to changing dynamics within the Indian family. Marriage is traditionally seen as means for procreation but there are slow perception changes among adolescents toward viewing it as a means of companionship.(4)

There is also a rise in the average age of marriage from 15 in the early 80s to 18 years among Indian women.(3) This has benefited women in terms of allowing them more time to mature physically and emotionally which again could influence their choice of marriage partner and expectations of family life. An increasing number of women are joining the workforce. This has brought about a change in the distribution of household and parenting responsibilities and requires more involvement from both spouses for which young adults may not be equipped. With couples migrating to cities for better job prospects, there is a decreased availability of the informal support provided by the extended family in the past, resulting in young couples having to develop greater problem-solving and coping skills on their own.

The goal of any kind of relationship education is to teach individuals/couples/families the skills and principles that research and theory suggest are associated with satisfying, supportive, and stable relationships. It aims at decreasing the risk of distress as well as prevents problems from developing.(20)

A marital and family life education program for young adults is essential in order to help them bridge the gap between what their families of origin considered as an ideal or happy marriage and what they now desire from a marriage partner and family life.
